# Predictive Value of Soluble CD14, Interleukin-6 and Procalcitonin For Lower Extremity Amputation in People with Diabetes with Foot Ulcers: A Pilot Study

**DOI:** 10.12669/pjms.303.4575

**Published:** 2014

**Authors:** Ahmet Karakas, Erol Arslan, Tolga Cakmak, Ibrahim Aydin, E.Ozgur Akgul, Seref Demirbas

**Affiliations:** 1 Ahmet Karakas, Assistant Professor, Department of Infectious Disease and Clinical Microbiology, Gulhane Military Medical Academy and School of Medicine, Ankara, Turkey.; 2 Dr. Erol Arslan, Assistant Professor, Department of Medicine, Gulhane Military Medical Academy and School of Medicine, Ankara, Turkey.; 3 Dr. Tolga Cakmak,Department of Aerospace Medicine, Military Hospital, Eskisehir, Turkey.; 4 Dr. Ibrahim Aydin, Department of Biochemistry, Sarikamis Military Hospital, Agri, Turkey.; 5 E.Ozgur Akgul, Associate Professor, Department of Biochemistry, Gulhane Military Medical Academy and School of Medicine, Ankara, Turkey.; 6 Seref Demirbas, Assistant Professor, Department of Medicine, Gulhane Military Medical Academy and School of Medicine, Ankara, Turkey.; 7 Senol Yildiz, Professor, Department of Undersea and Hyperbaric Medicinem Gulhane Military Medical Academy and School of Medicine, Ankara, Turkey.

**Keywords:** Amputation, Diabetic foot ulcer, Interleukin-6, Procalcitonin, Soluble CD14

## Abstract

***Objective: ***The aim of this pilot study was to determine clinical and laboratory factors that predict amputation surgery and to evaluate the predictive value of soluble CD14 (sCD14), interleukin-6 (IL-6), and procalcitonin (PCT) in patients with diabetic foot ulcers (DFUs).

***Methods: ***Twenty-seven (20 males, 7 females) Diabetic Foot Ulcers (DFU) patients admitted to our department were consecutively enrolled. The patients’ demographics and wound characteristics were noted. IL-6, PCT, and sCD14 were measured at admission.

***Results: ***Six of the 27 patients (22%) eventually underwent lower extremity amputation. Compared to the non-amputation group, a previous history of amputation (*p*=0.017), the presence of gangrene (*p*=0.044), the Wagner grade (*p*=0.011), the IL-6 concentration (*p*=0.018), the white blood cell count (WBC) (*p*=0.036), and the erythrocyte sedimentation rate (ESR) (*p*=0.042) were significantly high in the amputation group. However, the sCD14 and PCT concentration were not significantly different.

***Conclusion: ***We have shown for the first time that IL-6 may have predictive value for lower extremity amputation in patients with DFU. Further studies are needed to confirm its predictive value in this patient group.

## INTRODUCTION

Foot problems are among the most serious complications of diabetes mellitus. Twenty five percent of people with diabetes will develop a foot ulcer sometime during their life.^1^ The annual incidence of diabetic foot ulcers (DFUs) ranges from 2% to 32%, depending on the patients’ risk category, which was previously described by the American Diabetes Association.^2^^-^^4^ A significant number of DFUs become infected, and amputation is required in almost 20% of these patients.^5^

Proper management of DFU is crucial to reduce amputation rates in patients with diabetes. Predictive factors of limb loss in those with diabetes may help us to optimize their treatment. A number of studies have investigated clinical and laboratory findings as predictors of lower extremity amputations in patients with DFUs.^6^ These factors include age, male gender, presence of gangrene, high white blood cell count (WBC), anemia, poor glycemic control, and peripheral arterial disease.^6^^-^^   8^

There is still a need for new markers to evaluate the treatment response and to predict those patients who will eventually undergo amputation. In a recent study, Altay et al. suggested that interleukin-6 (IL-6), an indicator of the early response to inflammation and trauma, may be used in the diagnosis and follow up of patients with diabetic foot infections (DFIs).^9^ They found that the serum IL-6 level was significantly reduced after two weeks of treatment in patients whose wounds healed, whereas levels of IL-6 did not change in patients whose wounds did not heal.^9^ Procalcitonin (PCT) is another novel diagnostic marker in DFIs.^10^ Similar to IL-6, PCT levels decreased with treatment only in patients whose wounds healed.^9^ However, none of these markers were tested as predictors of amputation in patients with DFUs.

CD14, a highly specific receptor for lipopolysaccharides, is expressed on the surface of monocytes/macrophages.^11^ CD14 is found in two forms: membrane bound and soluble (sCD14).^12^ The normal range of the serum concentration of sCD14 in healthy adult volunteers was 1.5–5 mg/L.^13^ The serum concentration of sCD14 increased in sepsis and was associated with high mortality.^14^ To the best of our knowledge, the serum level of sCD14 in patients with DFUs has not been investigated before.

The aim of this pilot study was to determine clinical and laboratory factors that predict amputation in patients with DFUs. In addition, the serum concentration of sCD14 was measured in patients with DFUs for the first time in the literature. We also evaluated the predictive value of sCD14, IL-6, and PCT, along with other biochemical markers, in patients with DFUs.

## METHODS

***Patients: ***Patients with diabetes admitted to our department for nonhealing foot ulcers were consecutively enrolled in the study. Patients with other infectious diseases, those receiving antibiotherapy at admission, those who had undergone surgery in the previous six weeks, and those receiving immunosuppressive treatment were excluded. The patients’ demographics and wound characteristics were noted. Wound infection was diagnosed clinically by the presence of purulent secretions or at least two of the following symptoms: erythema, swelling, warmth, pain, or tenderness. Wounds were graded according to Wagner’s classification.^15^ All the patients received standard multidisciplinary treatment for DFI. This included glycemic regulation, wound care, antibiotherapy, and other adjunctive treatments, such as hyperbaric oxygen therapy, and negative pressure wound closure. Appropriate antibiotic treatment, which was chosen according to the results of wound cultures, was initiated. Off-loading of pressure points, daily wound dressing, and wound debridement were implemented when needed. The clinical progress of the patient was followed during daily visits, and wound images were taken regularly to evaluate the response to treatment. The Ethics Committee of Gulhane Military Medical Academy approved the study protocol, and all participants gave informed consent before enrollment.

***Laboratory Measurements: ***Blood samples, which were drawn for routine biochemical tests at admission, were used. The hospital biochemistry laboratory measured hemoglobin, hemoglobinA1c, WBC, the erythrocyte sedimentation rate(ESR), and C-reactive protein(CRP). Serum samples from all patients were stored at -80°C until blinded measurement of IL-6, PCT, and sCD14. IL-6 levels were measured using an automated solid phase, enzyme-labelled chemiluminescent immunometric assay (IMMULITE 2000, Siemens Healthcare Diagnostics, GmbH, Eschborn, Germany). Interassay coefficients of variation as determined in our laboratory were, depending on the sample concentration, between 3.9 and 14.3% for IL-6. The serum PCT concentration was measured using the Human Procalcitonin ELISA kit (RayBiotech, Inc. Norcross, GA). Serum sCD14 levels were quantified using the RayBio® Human CD14 ELISA Kit (RayBiotech, Inc., Norcross, GA) and performed according to the manufacturer’s protocol. All samples were measured in duplicate.

***Statistical Analysis: ***IBM’s SPSS Statistics 21.0 software program was used for statistical analyses. Data were presented as mean and standard deviation (SD). The Mann–Whitney *U*-test was used for continuous variables, and a chi-square test was used for categorical variables. The correlation between the biochemical variables was tested with Spearmen’s rho test. A *p* value less than 0.05 was regarded as statistically significant.

## RESULTS

In total, 27 patients were included into this study. There were 7 females and 20 males. The mean age of the patients was 62.4±12.1 years. The mean duration of diabetes mellitus was 17.3±5.9 years, and the mean HbA1c was 8.4±1.7%. Eleven patients had Wagner Grade 2, eight patients had Grade 3, and eight patients had Grade 4 wounds. Eighteen (66%) wounds were infected. Eventually, six patients (22%) underwent lower extremity amputation. Of these, three were below the knee amputation, and three were metatarsal amputations. 

The patients’ characteristics are presented in Table-I. The patients in the amputation and non-amputation groups were similar in terms of age, sex, diabetes duration, and HbA1c (Table-I). Compared to the non-amputation group, a previous history of amputation (42% vs. 100%, *p*=0.017) and the presence of gangrene (19% vs. 67%, *p*=0.044) were significantly high in the amputation group. The patients in the amputation group had higher Wagner grades (*p*=0.011). 

Blood samples were drawn in all patients at the initial examination to evaluate sCD14, IL-6, and PCT, along with commonly used biochemical markers (WBC, ESR, and CRP). Comparisons of the biochemical data of the amputation and the non-amputation group are presented in Table-II. Compared to the non-amputation group (9.9±9.3 pg/ml), the serum IL-6 concentration was significantly high in the amputation group (20.8±12.3 pg/ml) (*p*=0.018). However, the sCD14 and PCT concentration were not significantly different in the amputation and non-amputation groups (Table II). The WBC and the ESR, but not CRP, were significantly high in the amputation group compared to the non-amputation group (Table-II). Analysis of the correlation between sCD14, IL-6, PCT, and the infection markers revealed a significant correlation only between the IL-6 concentration and the ESR level (*r*=0.497, *p*=0.008).

## DISCUSSION

In this study, we investigated the predictive value of clinical and laboratory findings in DFUs. We found that a previous history of amputation, a higher Wagner grade, the presence of gangrene, and higher WBC, ESR, and IL-6 are associated with lower extremity amputation in patients with DFUs.

Lower extremity amputation is associated with subsequent amputation in patients with diabetes.^16^ A previous history of amputation was highly significant in predicting limb loss in our patients. All the patients who underwent amputation had a history of previous amputation. However, sex, age, and duration of diabetes were not associated with amputation in this study.

Among wound characteristics, a higher Wagner grade and the presence of gangrene were significantly associated with amputation in our patients. Similarly, Sun et al. found that patients with Wagner Grade 3 and 4 wounds had a 13.1 times higher risk for lower extremity amputation compared to patients with Wagner Grade 1 and 2 wounds.^17^ Almost, 50% of patients with gangrene underwent amputation in our study. In accordance with our findings, Aziz et al. found that a patient with gangrene has 5.6-fold increased risk of major amputation.^6^ Interestingly, the presence of wound infection was not associated with amputation in our study. Pittet et al. found that deep infection and osteomyelitis also predict amputation in DFU.^18^ In the present study, the WBC and ESR levels, which may represent the severity of an infection, were significantly higher in the amputation group. We think that the severity of infection is a better predictor of limb loss in DFU than the presence of infection.

The WBC and ESR are the most commonly used biochemical infection markers in DFUs. Aziz et al. found that a WBC≥15.0×10^9^/L and an ESR≥100mm/h are significant predictive factors for limb loss.^6^ We also found that the WBC and ESR levels were significantly high in the amputation group (Table-II). However, CRP was not statistically different in the amputation and the non-amputation groups.

IL-6 is an indicator of early response to inflammation and trauma. Monocytes, macrophages, fibroblasts, endothelial cells, keratinocytes, mast cells, T cells, and some tumor cells have the capacity to produce IL-6.^19^ Serum IL-6 levels increase in a wide variety of inflammatory processes, such as sepsis, autoimmune diseases, lymphoma, AIDS, alcoholic liver disease, infections, and acute graft-versus-host disease. Tuttolomondo et al. studied IL-6 in people with diabetes with or without foot ulcers and found higher levels of IL-6 in the DFU group. They proposed that an increase in the level of IL-6 is an indicator of anti-inflammatory activation in patients with DFU.^20^ Altay et al. measured serum levels of IL-6 during the course of DFI.^9^ They found that IL-6 significantly decreased after two weeks in patients whose wounds healed, whereas it did not change in those whose wounds did not heal.^9^ They suggested that IL-6 could show the treatment response in DFI. In our study, the serum concentration of IL-6 was 14.15±13.94 pg/ml, which is higher than the normal serum concentration of IL-6 (˂5.9 pg/mL).^21^ We also found that the IL-6 concentration was significantly higher in the amputation group compared to the non-amputation group (Table-II). We think that the serum IL-6 level can be used in the follow-up of patients with DFU. Further studies are needed to confirm the predictive value of IL-6 for lower extremity amputation in patients with DFU.

We also investigated serum levels of PCT in our patients. PCT, a 116-amino acid peptide, is emerging as a new serum marker of infection. PCT has been shown to increase in DFI.^10^^,^^22^ Altay et al. recommended that PCT can be used in the diagnosis and follow-up of patients with DFI.^9^ However, PCT failed to predict amputation in the patients with DFUs in this study. The serum concentration of PCT was similar in the patients who underwent amputation and who did not (Table-II).

To the best of our knowledge, this is the first study to determine the serum concentration of sCD14 in patients with DFUs. The normal range of serum concentration of sCD14 in healthy adult volunteers is 1.5–5 mg/L.^13^ We found that the sCD14 levels (5.84±3.13pg/ml) in our patients were within the normal range (<6 pg/ml). It was reported that sCD14 is elevated in inflammatory diseases and that it is a valuable marker in monitoring the response of patients to treatment.^23^ It was also shown to increase in type 2 diabetes mellitus patients, with the elevation attributed to damaged endothelial cells.^24^ A recent investigation demonstrated that CD14 modulates adipose tissue inflammatory activity and insulin resistance.^25^ Most of our patients had localized infections, which would not result in a systematic response. We think that this likely explains the lack of elevation in sCD14. We conclude that sCD14 has no value as a prognostic marker in patients with DFUs.

There are some limitations of our study. Neuropathy was not evaluated in our patients. Neuropathy is a known risk factor for amputation in patients with diabetes. As the primary aim of this study was to evaluate the predictive value of sCD14, IL-6, and PCT, the lack of a neuropathy score does not influence our results. The sample size is another limitation, as it precluded multivariate analyzes of independent predictors of amputation. The current study was a pilot study, and our findings should be tested in a larger prospective study.

**Table-I:** Demographic features and wound characteristics of the patients. Data were presented as mean ± SD or n (%).

		*Amputation*		
		*No*	*Yes*	*p*
n		21	6	
Age (y)		61.2±12.5	66.5±10.9	0.289
Sex (M/F)		16/5	4/2	0.633
Diabetic age (y)		17.0±6,5	18.6±3.2	0.441
DM > 20 years		9 (42%)	5 (83%)	0.098
HbA1c (%)		8.4±1.7	8.4±1.9	0.967
Wagner	Grade 2	11 (53%)	-	0.011
	Grade 3	6 (28%)	2 (33%)	
	Grade 4	4 (19%)	4 (67%)	
Gangrene		4 (19%)	4 (67%)	0.044
Wound infection		14 (67%)	4 (67%)	0.677
Previous history of amputation		9 (42%)	6 (100%)	0.017
Peripheral arterial disease		9 (42%)	5 (83%)	0.098

**Table-II:** Comparison of amputation and non amputation group in terms of biochemical data

	*Amputation*		
	*No*	*Yes*	*p*
n	21	6	
Haemoglobin (g/dl), mean±SD	13.0±1.7	12.0±2.5	0.316
White Blood Cell Count (10^3^/mm^3^), mean±SD	8.2±2.4	10.8±2.7	0.036
ESR (mm/h), mean±SD	41.3±24.9	73.0±36,5	0.042
C-Reactive Protein (mg/L), mean±SD	25.6±38,1	23.8±19.2	0.550
Interleukin 6 (pg/ml), mean±SD	9.7±9.3	20.8±12.3	0.018
sCD14 (pg/ml), mean±SD	5.83±3.41	5.88±2.07	0.932
Procalcitonin (pg/ml), mean±SD	0.079±0.079	1.15±2.65	0.157

*ESR:* *Erythrocyte Sedimentation Rate, SD: Standart deviation, sCD14: Soluble CD14*

## CONCLUSION

In this study, sCD14 and PCT failed to predict patients who will eventually require amputation. The concentration of IL-6, the WBC, and the ESR at admission were associated with amputation in patients with DFUs. Clinical features, a previous history of amputation, a higher Wagner grade, and the presence of gangrene were also associated with amputation in our patients. Further studies are needed to confirm the predictive value of IL-6 for lower extremity amputation in patients with DFUs.

## Authors Contributions:

**AK & SY:** Designed the study and prepared the final manuscript.

**EA, TC, I** **A & EOA: **Did data collection and manuscript writing.

**SD: **Did review and final approval of manuscript.

**AK:** Takes the responsibility and is accountable for all aspects of the work in ensuring that questions related to the accuracy or integrity of any part of the work are appropriately investigated and resolved.
